# Supplementary material for: Connective tissue inspired elastomer-based hydrogel for artificial skin via radiation-indued penetrating polymerization
Source: Nat Commun. 2024 Jan 20;15:636. doi: 10.1038/s41467-024-44949-1 (PMC10799914; doi:10.1038/s41467-024-44949-1)
Supplement: Supplementary file 1 — Supplementary Information [file 41467_2024_44949_MOESM1_ESM.pdf]

Supplementary information

Connective Tissue Inspired Elastomer-based Hydrogel for Artificial Skin via Radiation-indued Penetrating Polymerization

Yuan Tian[1], Zhihao Wang[1], Shuiyan Cao[2], Dong Liu[3], Yukun Zhang[1], Chong Chen[1], Zhiwen Jiang[1,4], Jun Ma[1,4], Yunlong Wang*[1]*

[1]College of Materials Science and Technology, Nanjing University of Aeronautics and Astronautics Nanjing 211106, P. R. China;

[2]College of Physics, MIIT Key Laboratory of Aerospace Information Materials and Physics, Nanjing University of Aeronautics and Astronautics, Nanjing, 211106, China

[3]Key Laboratory of Neutron Physics and Institute of Nuclear Physics and Chemistry, China Academy of Engineering Physics, Mianyang, 621999, China

[4]School of Nuclear Science and Technology, University of Science and Technology of China, Hefei, Anhui230026, China

Corresponding author: wylong@nuaa.edu.cn; junma@nuaa.edu.cn

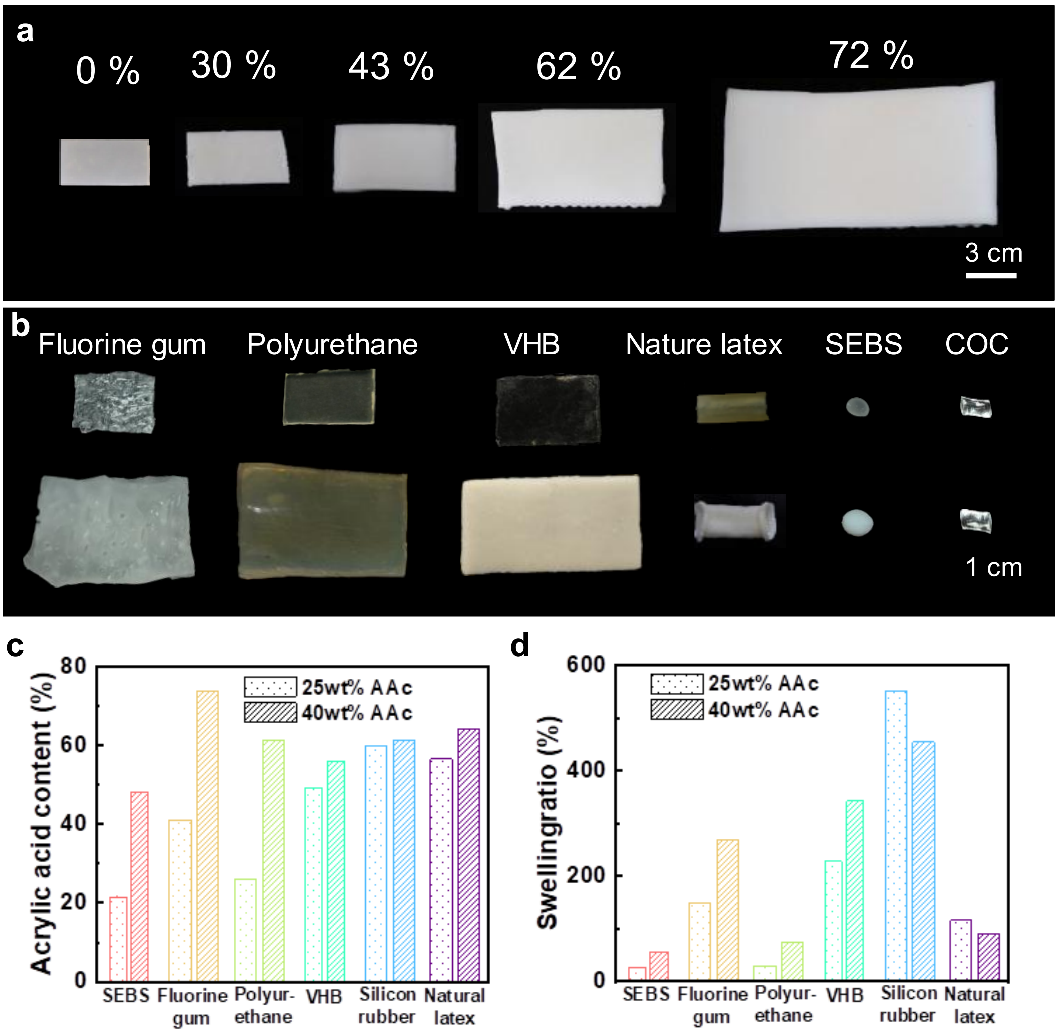

Supplementary Figure 1: (a) Full water-swollen CEBH with different acrylic acid content. (b) Comparison of different materials before (upper) and after (lower, swollen in water) modification by radiation induced penetrating grafting. (c, d) AC and water swelling ratio of different materials modified by radiation induced penetrating grafting of acrylic acid.

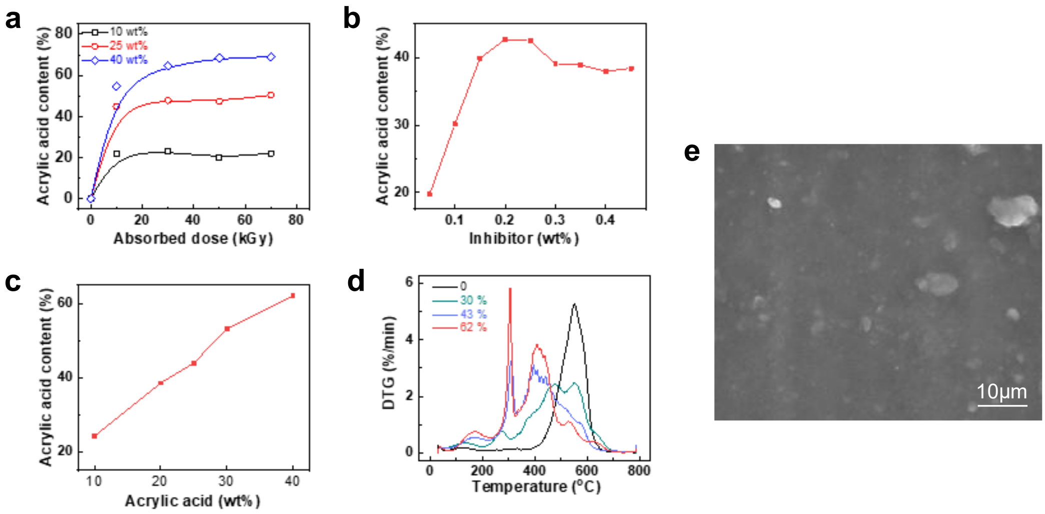

Supplementary Figure 2: (a) AC of grafted samples with different absorbed dose and AA concentration (with 0.25 wt% Mohr's salt). (b) Effect of inhibitor (Mohr's salt) percentage on the AC of grafted sample (with monomer concentration of 25% and absorbed dose of 10 kGy). (c) The influence of acrylic acid concentration on the grafting ratio (with absorbed dose of 30 kGy, and 0.25 wt% Mohr's salt). (d) Differential thermogravimetric curve of CEBH with different AC. (e) The SEM image of CEBH, the AC is 30%.

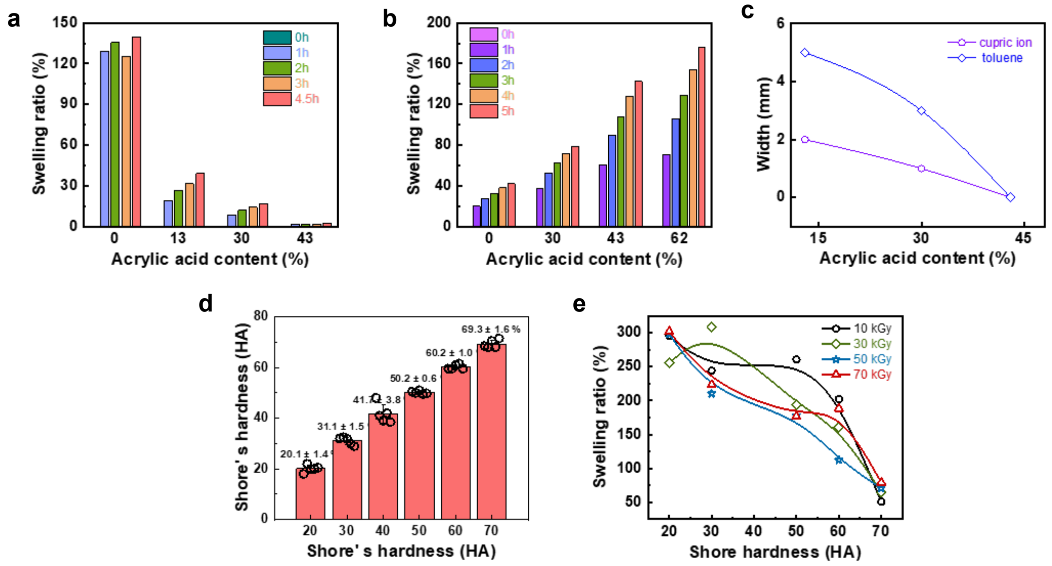

Supplementary Figure 3: The swelling ratio of CEBH with different AC in (a) toluene and (b) water. (c) For the relationship between modified silica rubber width and AC in different solution. (d) The actual hardness of silicone rubber with different hardness was measured by a hardness tester (n = 5 parallel tests for each sample). (e) The swelling ratio of CEBH prepared from silicon rubber with different Shore hardness.

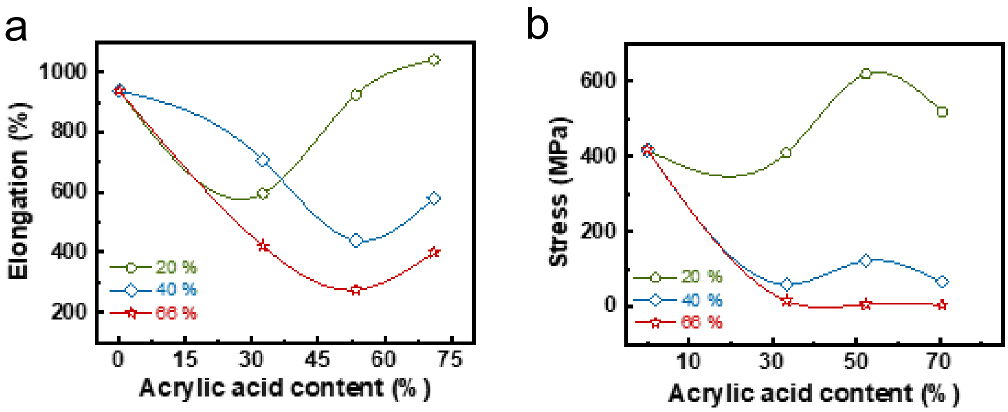

Supplementary Figure 4: (a) Tensile length, (b) Maximum compressive stress of CEBH with different AC and water content (20%, 40% and 60%).

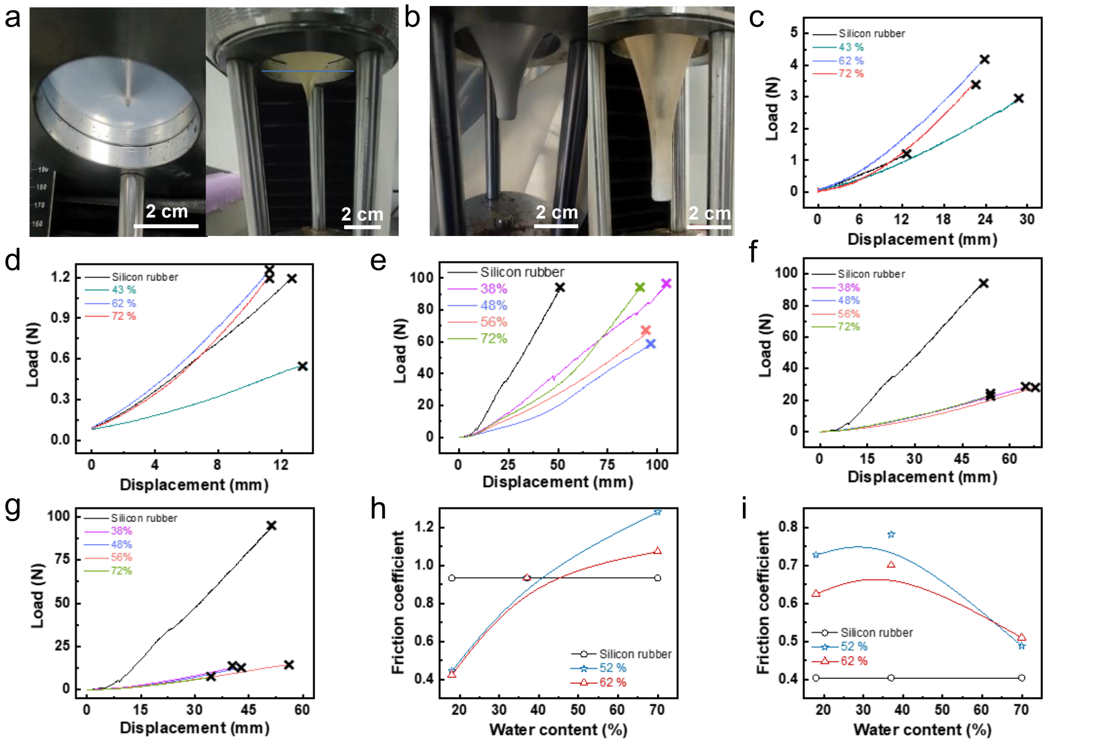

Supplementary Figure 5: In the puncture experiment, the comparison before (left) and after (right) modification ((a) needle diameter 1 mm, (b) needle diameter 10 mm). (c d) The force-displacement curve obtained by puncture test of CEBH with different AC (silicon rubber, 43%, 62%, and 72%) and different water content (40% for c and 66% for d), the diameter of the needle is 1 mm. (e-g) The force-displacement curve obtained by puncture test of CEBH with different AC (silicon rubber, 38%, 48%, 56%, and 72%) and different water content (20% for e, 40% for f and 66% for g), the diameter of the needle is 10mm. (h, i) The friction coefficient-water of CEBH with different AC (0%, 52% and 62%) under (h) dry and (i) wet conditions.

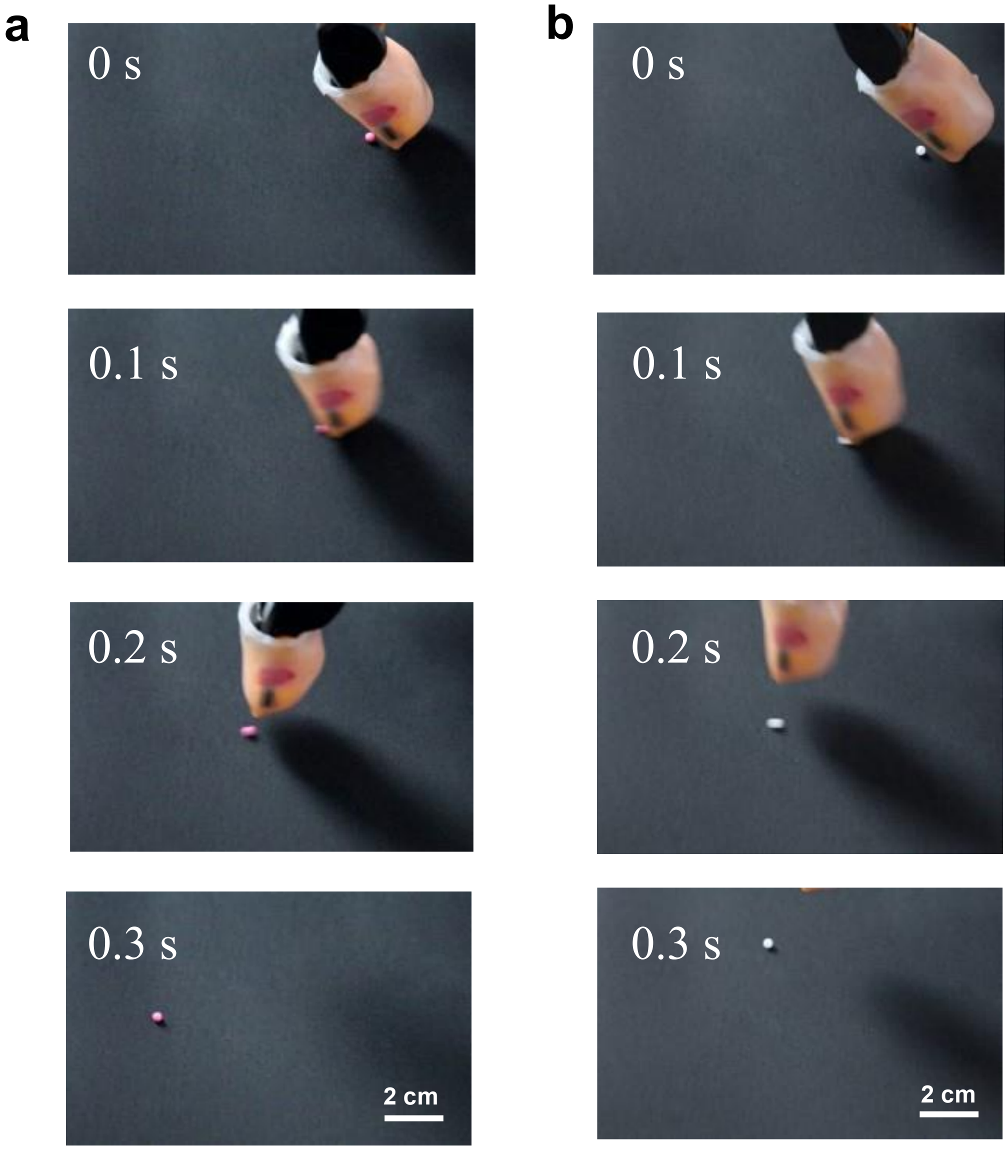

Supplementary Figure 6: Flicking polystyrene foam beads using CEBH covered robot arm. (a) acrylic material (d = 3mm) and (b) POM material (d = 3mm).

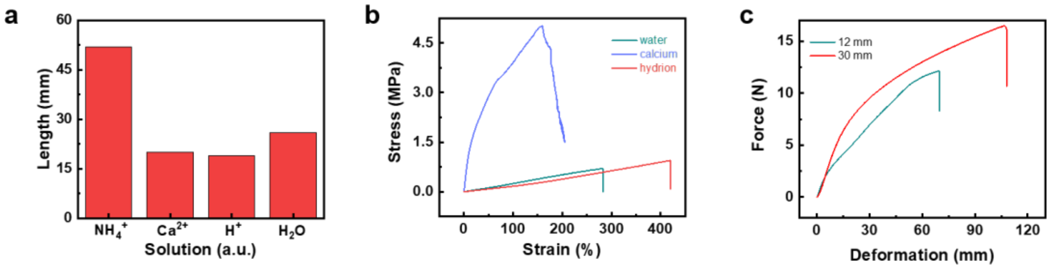

Supplementary Figure 7: (a) Length of CEBH films immersed in different ion solutions and water. (b) Strain-stress curve of CEBH film after being immersed in different ion solutions. (c) The strain-deformation curve of load bearing capacity test of CEBH soaked in Ca^{2+} solution, with different contact length (12 mm and 30 mm).

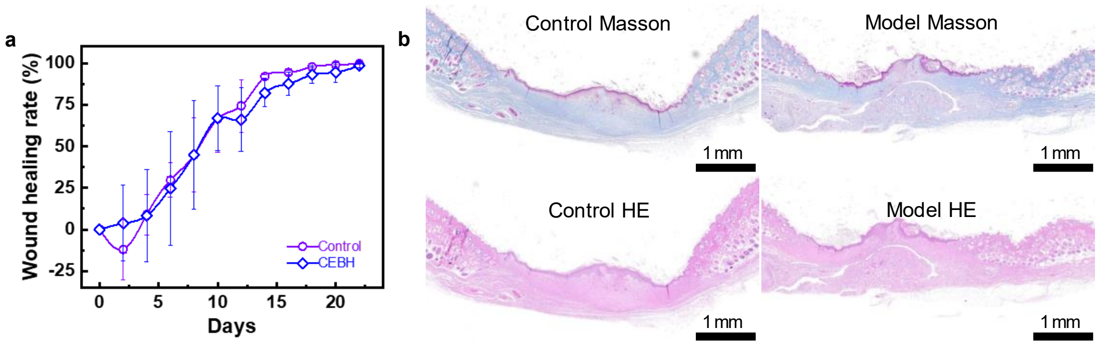

Supplementary Figure 8: In-vivo wound healing experiment results. (a) Wound closure rates on days 0, 2, 4, 6, 8, 10, 12, 14, 16, 18, 20 and 22 (datas collected from n = 3 independent experiment animals). No significant diffrentce were found between healing rates of control and model groups were found. (p = 0.2020, two-side t-test) (b) Masson's and HE staining of wound sections obtained from control and model groups on day 22.

Supplementary Table 1 Comparison of mechanical properties of CEBH, DN hydrogel, Human skin.

Hydrogel	Yong's modulus (MPa)	Friction Coefficient	Compressive Resitance (MPa)
CEBH	0.048-3.2	0.36-1.3	5.7-620
BC-PVA-PAMPS[1]	155-227	0.06	17.3-23
Agar-PAAm[2]	0.08	--	38
PVDT-PEGDA[3]	0.12	--	6
HA-SS-PEG[4]	0.01-0.05	--	0.08-0.32
Agarose hydrogel[5]	--	0.005-0.09	1
Highly entangled PAAm hydrogel[6]	35	0.0067	--
PAAN[7]	0.006-0.07	--	0.54-8.53
Chitosan-gelatin-phytate[8]	0.03-2.47	--	35.7-64
Human skin	0.1-2	0.4-0.8	0.3

Supplementary Table 2 Comparison of anti-puncture performance of different hydrogels.

Hydrogel	Load（N）	Needle diameter（mm）
BRC[9]	1.06*	0.3 (using a needle)
alginate hydrogels[10]	1.2	\
SA-AAm[11]	12	10
PAAm[12]	0.5	1
PVA/SA/Gly hydrogel[13]**	57	1
s-BNCH[14]***	50	3 (using a needle)
This work	96 (max)	10
	7.2 (max)	1

* Calculated from the needle diameter and pressure reported (15 MPa); ** Hydrogel composites laminated with aramid fabric; *** Montmorillonite reinforeced hydrogel.

References

1. Yang F, *et al.* A Synthetic Hydrogel Composite with the Mechanical Behavior and Durability of Cartilage. **30**, 2003451 (2020).

2. Chen Q, Zhu L, Zhao C, Wang Q, Zheng J. A Robust, One-Pot Synthesis of Highly Mechanical and Recoverable Double Network Hydrogels Using Thermoreversible Sol-Gel Polysaccharide. **25**, 4171-4176 (2013).

3. Dai X, *et al.* A Mechanically Strong, Highly Stable, Thermoplastic, and Self-Healable Supramolecular Polymer Hydrogel. **27**, 3566-3571 (2015).

4. Ren H, Zhang Z, Cheng X, Zou Z, Chen X, He C. Injectable, self-healing hydrogel adhesives with firm tissue adhesion and on-demand biodegradation for sutureless wound closure. **9**, eadh4327 (2023).

5. Geurds L, Xu Y, Stokes JR. Friction of lubricated hydrogels: Influence of load, speed and lubricant viscosity. *Biotribology* **25**, 100162 (2021).

6. Kim J, Zhang G, Shi M, Suo Z. Fracture, fatigue, and friction of polymers in which entanglements greatly outnumber cross-links. *Science (New York, NY)* **374**, 212-216 (2021).

7. Su G, *et al.* Balancing the mechanical, electronic, and self-healing properties in conductive self-healing hydrogel for wearable sensor applications. *Materials horizons* **8**, 1795-1804 (2021).

8. Xu L, Wang C, Cui Y, Li A, Qiao Y, Qiu D. Conjoined-network rendered stiff and tough hydrogels from biogenic molecules. **5**, eaau3442 (2019).

9. Li R, *et al.* Breakage-resistant hydrogel electrode enables ultrahigh mechanical reliability for triboelectric nanogenerators. *Chemical Engineering Journal* **454**, 140261 (2023).

10. Kaklamani G, Cheneler D, Grover LM, Adams MJ, Anastasiadis SH, Bowen J. Anisotropic dehydration of hydrogel surfaces. *Progress in Biomaterials* **6**, 157-164 (2017).

11. Jiang Z, Liu J, Li Y, Kang G. Indentation and puncture of double-network tough hydrogel membranes. *Polym Test* **116**, 107782 (2022).

12. Fakhouri S, Hutchens SB, Crosby AJ. Puncture mechanics of soft solids. *Soft Matter* **11**, 4723-4730 (2015).

13. Wang Q, Wang S, Chen M, Wei L, Dong J, Sun R. Anti-puncture, frigostable, and flexible hydrogel-based composites for soft armor. *Journal of Materials Research and Technology* **21**, 2915-2925 (2022).

14. Huang Y*, et al.* Aligned Porous and Anisotropic Nanocomposite Hydrogel with High Mechanical Strength and Superior Puncture Resistance by Reactive Freeze-Casting. *Chemistry of Materials* **35**, 5809-5821 (2023).
