# Supplementary material for: Connective tissue inspired elastomer-based hydrogel for artificial skin via radiation-indued penetrating polymerization
Source: Nat Commun. 2024 Jan 20;15:636. doi: 10.1038/s41467-024-44949-1 (PMC10799914; doi:10.1038/s41467-024-44949-1)
Supplement: Supplementary file 3 — Description of Additional Supplementary Files [file 41467_2024_44949_MOESM3_ESM.pdf]

Description of Additional Supplementary Files

Supplementary Movie 1: Compression and recovery of CEBH.

Supplementary Movie 2: Static puncture tests of unmodified silicone rubber and CEBH using needles with different diameters of 1 mm and 10 mm.

Supplementary Movie 3: Demonstration using a manipulator covered with CEBH grabbing an egg in the air.

Supplementary Movie 4: Demonstration using a manipulator covered with CEBH grabbing an egg underwater.

Supplementary Movie 5: Demonstration of flipping different particles (POM, acrylic, PS foam) with CEBH and the adhesion of PS foam particles on traditional PS foam particles.

Supplementary Movie 6: Demonstration of controlling liquid flowing by an ions triggered CEBH switch using Ca^{2+} ions.

Supplementary Movie 7: Demonstration of Ca^{2+} triggered shrinking of CEBH wound healing film.
