# Supplementary material for: Connective tissue inspired elastomer-based hydrogel for artificial skin via radiation-indued penetrating polymerization
Source: Nat Commun. 2024 Jan 20;15:636. doi: 10.1038/s41467-024-44949-1 (PMC10799914; doi:10.1038/s41467-024-44949-1)
Supplement: Supplementary file 11 — Reporting Summary [file 41467_2024_44949_MOESM11_ESM.pdf]

nature portfolio

Corresponding author(s): Wang Yunlong

Last updated by author(s): Jan 10, 2024

Reporting Summary

Nature Portfolio wishes to improve the reproducibility of the work that we publish. This form provides structure for consistency and transparency in reporting. For further information on Nature Portfolio policies, see our Editorial Policies and the Editorial Policy Checklist.

Statistics

For all statistical analyses, confirm that the following items are present in the figure legend, table legend, main text, or Methods section.

n/a	Confirmed	
☐	☒	The exact sample size (*n*) for each experimental group/condition, given as a discrete number and unit of measurement
☐	☒	A statement on whether measurements were taken from distinct samples or whether the same sample was measured repeatedly
☐	☒	The statistical test(s) used AND whether they are one- or two-sided<br>*Only common tests should be described solely by name; describe more complex techniques in the Methods section.*
☒	☐	A description of all covariates tested
☒	☐	A description of any assumptions or corrections, such as tests of normality and adjustment for multiple comparisons
☐	☒	A full description of the statistical parameters including central tendency (e.g. means) or other basic estimates (e.g. regression coefficient) AND variation (e.g. standard deviation) or associated estimates of uncertainty (e.g. confidence intervals)
☐	☒	For null hypothesis testing, the test statistic (e.g. *F*, *t*, *r*) with confidence intervals, effect sizes, degrees of freedom and *P* value noted<br>*Give P values as exact values whenever suitable.*
☒	☐	For Bayesian analysis, information on the choice of priors and Markov chain Monte Carlo settings
☒	☐	For hierarchical and complex designs, identification of the appropriate level for tests and full reporting of outcomes
☒	☐	Estimates of effect sizes (e.g. Cohen's *d*, Pearson's *r*), indicating how they were calculated

Our web collection on statistics for biologists contains articles on many of the points above.

Software and code

Policy information about availability of computer code

Data collection	NMR:TopSpin 3.6.1; FT-IR: OMNIC 8; OD: TECAN i-control; TG&DSC:Netzsch Proteus 4.2;
Data analysis	Microsoft Excel 2021; origin 2019; BerSANS ver 14-Aug-2014; MestRenova 14; Slideviewer v2. 5; Graphpad Prism (version 8.0);

For manuscripts utilizing custom algorithms or software that are central to the research but not yet described in published literature, software must be made available to editors and reviewers. We strongly encourage code deposition in a community repository (e.g. GitHub). See the Nature Portfolio guidelines for submitting code & software for further information.

Data

Policy information about availability of data

All manuscripts must include a data availability statement. This statement should provide the following information, where applicable:

- Accession codes, unique identifiers, or web links for publicly available datasets
- A description of any restrictions on data availability
- For clinical datasets or third party data, please ensure that the statement adheres to our policy

The authors declare that the data supporting the findings of this study are available within the article and its Supplementary Information. Source data are provided with this paper (DOI: 10.6084/m9.figshare.24041514). Additional data are available from the corresponding author upon request.

Research involving human participants, their data, or biological material

Policy information about studies with human participants or human data. See also policy information about sex, gender (identity/presentation), and sexual orientation and race, ethnicity and racism.

Reporting on sex and gender	Not applicable
Reporting on race, ethnicity, or other socially relevant groupings	Not applicable
Population characteristics	Not applicable
Recruitment	Not applicable
Ethics oversight	Not applicable

Note that full information on the approval of the study protocol must also be provided in the manuscript.

Field-specific reporting

Please select the one below that is the best fit for your research. If you are not sure, read the appropriate sections before making your selection.

[x] Life sciences [ ] Behavioural & social sciences [ ] Ecological, evolutionary & environmental sciences

For a reference copy of the document with all sections, see nature.com/documents/nr-reporting-summary-flat.pdf

Life sciences study design

All studies must disclose on these points even when the disclosure is negative.

Sample size	Sample sizes were based on a combination of the resource-equation method and prior laboratory experience, to ensure statistical and biological significance. One-way ANOVA with Tukey's multiple comparisons was used for multiple comparisons when more than two groups were compared, and two-tailed Student's t-test was used for two-group comparisons.
Data exclusions	No data were excluded.
Replication	All experiments were successfully replicated at least in 3 independent experiments.
Randomization	Experiment animals are assigned to control or model groups randomly. Cells were randomly assigned to different groups and performed independently.
Blinding	The investigators were blinded to the cell groups and mouse groups when collecting the results.

Reporting for specific materials, systems and methods

We require information from authors about some types of materials, experimental systems and methods used in many studies. Here, indicate whether each material, system or method listed is relevant to your study. If you are not sure if a list item applies to your research, read the appropriate section before selecting a response.

Materials & experimental systems

n/a	Involved in the study	
x		Antibodies
	x	Eukaryotic cell lines
x		Palaeontology and archaeology
	x	Animals and other organisms
x		Clinical data
x		Dual use research of concern
x		Plants

Methods

n/a	Involved in the study	
x		ChIP-seq
x		Flow cytometry
x		MRI-based neuroimaging

Eukaryotic cell lines

Policy information about cell lines and Sex and Gender in Research

Cell line source(s)	L929 (mouse fibroblast) cell lines were obtained from the Cell Bank of the Chinese Academy of Sciences (Shanghai, China).
Authentication	L929 cells were purchased from Cyperus Biologicals (cat. GNM28, authenticated by Cell Bank of the Chinese Academy of Science). After receiving cell lines, cell identity was confirmed by morphological assessment under the microscope.
Mycoplasma contamination	The cell lines were regularly tested for mycoplasma contamination, and no mycoplasma contamination was found.
Commonly misidentified lines (See ICLAC register)	No commonly misidentified lines were used.

Animals and other research organisms

Policy information about studies involving animals; ARRIVE guidelines recommended for reporting animal research, and Sex and Gender in Research

Laboratory animals	The wound healing experiments use 8 week aged female mice (BALB/c) and have been approved by the Animal Ethics Committee of Yanxuan Biotechnology (Hangzhou) Co., Ltd. (Approval No. YXSW2309269698).
Wild animals	This study didn't involve wild animals.
Reporting on sex	All mice used in wound healing experiments are female, for sex has been reported to have affect on the wound healing, and we found that female lab animals were widely used in similar experiments.
Field-collected samples	This study didn't involve field collected samples.
Ethics oversight	Animal experiments approved by the Animal Ethics Committee of Yanxuan Biotechnology (Hangzhou) Co., Ltd. (Approval No. YXSW2309269698). Other experiments in this work does not involve human or animal experiments.

Note that full information on the approval of the study protocol must also be provided in the manuscript.
